# High prevalence of dyslipidaemia subtypes and their associated personal and clinical attributes in Malaysian adults: the REDISCOVER study

**DOI:** 10.1186/s12872-021-01956-0

**Published:** 2021-03-23

**Authors:** Mohamed-Syarif Mohamed-Yassin, Noorhida Baharudin, Aqil Mohammad Daher, Najmin Abu Bakar, Anis Safura Ramli, Suraya Abdul-Razak, Nor-Ashikin Mohamed Noor Khan, Mariam Mohamad, Khalid Yusoff

**Affiliations:** 1 Department of Primary Care Medicine, Faculty of Medicine, Universiti Teknologi MARA, Selayang Campus, Jalan Prima Selayang 7, 68100 Batu Caves, Selangor, Malaysia; 2 Department of Community Medicine, School of Medicine, International Medical University, Bukit Jalil, 57000 Kuala Lumpur, Malaysia; 3 Centre for Translational Research and Epidemiology (CenTRE), Faculty of Medicine, Universiti Teknologi MARA, Jalan Hospital, 47000 Sungai Buloh, Selangor, Malaysia; 4 Institute of Pathology, Laboratory and Forensic Medicine (I-PPerForM), Universiti Teknologi MARA, Sungai Buloh Campus, Jalan Hospital, 47000 Sungai Buloh, Selangor, Malaysia; 5 Department of Physiology, Faculty of Medicine, Universiti Teknologi MARA, Sungai Buloh Campus, Jalan Hospital, 47000 Sungai Buloh, Selangor, Malaysia; 6 Department of Public Health Medicine, Faculty of Medicine, Universiti Teknologi MARA, Sungai Buloh Campus, Jalan Hospital, 47000 Sungai Buloh, Selangor, Malaysia; 7 Faculty of Medicine and Health Sciences, UCSI University, No.1, Jalan Menara Gading, UCSI Heights, Taman Connaught, 56000 Cheras, Kuala Lumpur, Malaysia; 8 Faculty of Medicine, Universiti Teknologi MARA, Sungai Buloh Campus, Jalan Hospital, 47000 Sungai Buloh, Selangor, Malaysia

**Keywords:** Dyslipidaemia, LDL-c, HDL-c, Non-HDL-c, Triglycerides, Hypercholesterolaemia, Malaysia, Prevalence

## Abstract

**Background:**

Dyslipidaemia refers to lipid abnormalities consisting of either one or any combination of the following: elevated total cholesterol (TC), elevated low-density lipoprotein cholesterol (LDL-c), elevated triglycerides (TG), and low high-density lipoprotein cholesterol (HDL-c). The prevalence of hypercholesterolaemia is steadily increasing in Malaysia. However, data on the prevalence of dyslipidaemia subtypes among Malaysians are lacking. This is important as it may have implications for preventive and management strategies for this increasing public health challenge. This study is aimed at determining the prevalence of dyslipidaemia subtypes and their associated personal and clinical attributes in Malaysians.

**Methods:**

REDISCOVER, a prospective study, enrolled 11,288 adults where sociodemographic data, anthropometric and blood pressure measurements, fasting lipid profile and glucose, and history of diabetes, hypertension, and smoking were obtained. The cross-sectional analytic sample presented in this article comprised 10,482 participants from baseline recruitment. The data was analysed by descriptive statistics and multivariable logistic regression.

**Results:**

The overall prevalence of elevated TC, elevated LDL-c, elevated TG, low HDL-c, and elevated non-HDL-c were 64.0% (95% CI 63.0–65.0), 56.7% (CI 55.7–57.7), 37.4% (CI 36.5–38.4), 36.2% (CI 35.2–37.1), and 56.2% (CI 55.3–57.2), respectively. Overweight, obesity, and central obesity were highly prevalent and significantly associated with elevated TC and all dyslipidaemia subtypes. Older age was associated with elevated TC, elevated LDL-c and elevated non-HDL-c. Hypertension was associated with elevated TC, elevated TG, and elevated non-HDL-c, while diabetes was associated with elevated TG and low HDL-c.

**Conclusions:**

Elevated TC and all dyslipidaemia subtypes are highly prevalent in Malaysia where increased body mass seems the main driver. Differences in the prevalence and associated personal and clinical attributes may facilitate specific preventive and management strategies.

## Background

Dyslipidaemia refers to lipid abnormalities consisting of either one or any combination of the following: elevated total cholesterol (TC), elevated low-density lipoprotein cholesterol (LDL-c), elevated triglycerides (TG), and low high-density lipoprotein cholesterol (HDL-c) [1]. Whilst TC, and particularly LDL-c are established as the most important cardiovascular risk factors, recent evidence suggests that non-high density lipoprotein cholesterol (non-HDL-c) is a more powerful cardiovascular (CV) risk predictor than LDL-c alone [2–4]. Non-HDL-c encompasses the total amount of atherogenic lipoproteins [very low-density lipoprotein (VLDL), VLDL remnants, intermediate-density lipoprotein (IDL), LDL-c and lipoprotein(a)] [1]. TG has also been considered to be an important CV risk factor either independently, or as a component of atherogenic dyslipidaemia, along with low HDL-c and elevated levels of small dense LDL particles [5, 6].

The World Health Organization (WHO) reported that the prevalence of hypercholesterolaemia were the highest in Europe (53.7%) and America (47.7%), while South East Asia (30.3%) and Africa (23.1%) had much lower prevalence [7]. However, Lin et al*.* reported marked prevalence differences between different Asia Pacific countries, ranging from 9% in Indonesia to 46.9% in the Philippines [8]. For high LDL-c, high TG, and low HDL-c, the prevalence ranges from 7.8 to 47.2%, 13.9 to 38.6%, and 10.1 to 71.3%, respectively [8].

In Malaysia, the National Health and Morbidity Surveys (NHMS) reported the increasing prevalence of hypercholesterolaemia from 20.7% in 2006 [9], 35.1% in 2011 [10] and 47.7% in 2015 [11]. Small scale studies have assessed the prevalence of dyslipidaemia in specific populations such as rural Malays [12] and male factory workers [13]. However, to the best of our knowledge, there has been no report on the prevalence of non-HDL-c in Malaysians. The current Malaysian dyslipidaemia management guideline recommends that LDL-c should be the primary treatment target [1]. It also suggests that non-HDL-c can be used as a secondary target for individuals with combined hyperlipidaemias, diabetes, metabolic syndrome, or chronic kidney disease [1]. Therefore, it is pertinent to establish the prevalence of all dyslipidaemia subtypes including high non-HDL-c in order to achieve individualised patient targets as recommended by the national guideline.

We report the prevalence of elevated total cholesterol and each dyslipidaemia subtype including high non-HDL-c in Malaysian adults aged ≥ 30 years from the REDISCOVER study. We also examine the relationship between these dyslipidaemia subtypes with personal and clinical attributes in this population.

## Methods

### Study design and population

The REDISCOVER (*Re*spon*d*ing to *I*ncrea*s*ing *C*ardi*ov*ascular Dis*e*ase P*r*evalence) study is an ongoing prospective study involving Malaysians aged 30 years and above, involving 22 rural and 18 urban communities from five states across Malaysia. The states involved are Selangor, Negri Sembilan, Pahang, Kelantan, and Sabah.

### Sampling methods: state and site selection

The five states were selected to ensure satisfactory representation of the major ethnic groups in Malaysia. In Peninsular Malaysia, the main ethnic groups are the Malays, Chinese and Indians. In Sabah (East Malaysia), the major ethnic groups are the Kadazan-Dusun, Bajau, and Murut. For this study, these groups from Sabah along with several other ethnic minorities were classified as the indigenous group. The other states selected (Selangor, Negri Sembilan and Pahang) have a robust mix of Malays, Chinese and Indians. Kelantan consists of a majority Malay populace, while Sabah represents the indigenous population.

### Sampling methods: subjects recruitment

A standardized method of recruitment was used. Announcements and invitation were made via local community leaders. Written invitation was issued for all household member above 30 years old to attend screening sessions at local community centres with an eight hour fast. The response rate for each site was between 60 to 70%. At the screening centres, the participants were screened for eligibility and an informed consent obtained. The study was approved by the institutional ethics committee [Ethics approval number: REC/UITM/2007(10)].

### Study procedures

All investigators and interviewers underwent training on the study procedures. A standard data collection form recorded the age, gender, ethnicity, educational attainment, smoking status, diabetes mellitus, hypertension, cardiovascular disease (CVD), and use of cholesterol-lowering medication. Education attainment was divided into four subgroups i.e. “no formal education”, “primary”, “secondary”, and “tertiary”. Primary education was defined as schooling from the ages of seven to 12 years old, while secondary education was schooling from the ages of 13 to 17 years old, and tertiary education attainment of college or university education. Population of ≥ 10,000 was classified as urban and those with < 10,000 as rural (Malaysian Population and Housing Census 2000) [14]. Smoking was classified as: 1. Current smokers were those who were currently smoking or, had smoked any tobacco products within the recent five years, 2. Non-smokers were those who had never smoked, 3. Ex-smokers were those who had quit smoking for more than five years. Diabetes was defined as fasting plasma glucose at ≥ 7.0 mmol/L and/or self-reported diabetes, and/or taking medications for diabetes in the past month. Cardiovascular diseases included ischaemic heart disease (IHD) and stroke that were reported by the participants.

Waist and hip circumferences were measured (to the nearest 0.1 cm) using a non-stretchable measuring tape while the participants stood in a relaxed position with arms by their side. Blood pressure was measured using the Omron automatic digital blood pressure monitors (Omron HEM-757) after a five-minute rest. Right arm BP measurements were taken on two occasions, two minutes apart while participants were seated, and a mean of the two readings was taken as the BP for the participant.

Participants were classified based on body mass index (BMI in kgm^−2^) into: Underweight < 18.5, normal 18.5–22.9, overweight 23–27.4, or obese ≥ 27.5 [15]. Abdominal or central obesity was defined as waist-hip ratio (WHR) of ≥ 0.90 and ≥ 0.85, in males and females respectively [16]. Hypertension was defined as mean systolic BP of ≥ 140 and/or mean diastolic BP ≥ 90 mmHg; or self-reported hypertension; or taking antihypertensive medications in the last month.

Fasting venous blood samples were collected for serum lipid profile [total cholesterol (TC), triglycerides (TG), high-density lipoprotein cholesterol (HDL-c), low-density lipoprotein cholesterol (LDL-c)] and plasma glucose. All variables, except for LDL-c were analysed using an automated clinical chemical analyzer (Cobas Integra 400 plus, Roche Diagnostic, Basel, Switzerland). LDL-c was calculated using the Freidewald equation (for TG ≤ 4.5 mmol/L) [1]. Non-HDL-c was calculated using the following equation TC—HDL-c (mmol/L) [1]. Dyslipidaemia is defined as either one or a combination of the following lipid levels [1]:

- TC > 5.2 mmol/L;
- LDL-c > 3.4 mmol/L;
- TG > 1.7 mmol/L;
- HDL-c < 1 in males; < 1.2 mmol/L in females;
- Non-HDL-c levels were considered high if values > 4.2 mmol/L [1].

### Data collection

The REDISCOVER study duration is 15 years and the baseline data were collected from 2007 to 2011. Data are being collected every three years. A total of 11,288 participants were recruited, with 806 participants who were taking cholesterol-lowering medication excluded from this analysis. The cross-sectional analytic sample presented in this article comprised 10,482 participants from baseline recruitment.

### Statistical analysis

Data were entered and analyzed using STATA software version 14 (StataCorp.TX). Missing data was automatically removed during analysis via pairwise deletion where cases without specific variable data were excluded from analysis of that variable only. Categorical variables were presented using frequencies and percentage, while numerical variables were presented using mean (± standard deviations [SD]). Chi-square test was used to compare categorical variables. Sensitivity analysis was done by comparing crude and age-adjusted prevalence. To estimate the crude and adjusted odds ratio for the factors associated with the subtypes of dyslipidaemia, simple and multiple regression models were utilized, and interactions were checked. Sample size was calculated for estimating prevalence of 0.5 with 0.03 margin of error and 95% CI was 964. To accommodate for design effect of 4 and non-response rate of 30%, the final sample size was 3855. A *p* value of less than 0.05 was considered statistically significant.

## Results

### Characteristics of the participants

10,482 participants were included in this analysis. The sociodemographic and clinical characteristics of the participants are shown in Table 1. The mean age was 52.7 (± 11.1) years. There were more females than males (56.7% vs. 43.3%). Malays were the predominant ethnicity (72.9%), and there was an almost equal split of urban and rural participants (50.2% vs. 49.8%). Almost thirty nine percent of the participants were overweight while 33.6% were obese. Forty six percent of the participants had hypertension, 15.1% had diabetes and 13.3% were current smokers.

**Table 1:** Sociodemographic and clinical characteristics (N = 10,482)

Sociodemographic and clinical characteristics	
Age (years) (± SD)	52.7 (± 11.1)
*Age groups (years) (n,%)*	
30–39	1207 (11.5)
40–49	3196 (30.5)
50–59	3277 (31.3)
≥ 60	2802 (26.7)
*Gender (n,%)*	
Male	4538 (43.3)
Female	5944 (56.7)
*Ethnicity (n,%)*	
Malay	7642 (72.9)
Chinese	1049 (10.0)
Indian	276 (2.6)
Indigenous	1515 (14.5)
*Education attainment (n*^*i*^*,%)*	
No formal education	1499 (15.8)
Primary school	2589 (27.3)
Secondary school	3628 (38.3)
Tertiary	1762 (18.6)
*Location (n,%)*	
Urban	5262 (50.2)
Rural	5220 (49.8)
*Smoking status (n*^*ii*^*,%)*	
Non-smoker	7431 (75.7)
Ex-smoker	1080 (11.0)
Current smoker	1307 (13.3)
*Diabetes mellitus (n,%)*	
No	8902 (84.9)
Yes	1580 (15.1)
*Hypertension (n,%)*	
No	5663 (54.0)
Yes	4819 (46.0)
*Self-reported cardiovascular disease (n,%)*	
No	10,039 (95.8)
Yes	443 (4.2)
*Body mass index (kg/m*^*2*^*) (n*^*iv*^*,%)*	
Underweight (< 18.5)	385 (3.9)
Normal (< 22.9)	2377 (24.0)
Overweight (23–27.4)	3814 (38.5)
Obese (> 27.4)	3326 (33.6)
*Waist-hip ratio (n*^*V*^*,%)*	
Normal	4303 (43.7)
Abdominal obesity (Male ≥ 0.90; female ≥ 0.85)	5539 (56.3)

n^i,ii,iii,iv,v^ are not equal to 10,482 due to missing values

Missing values: ^i^ 1004, ^ii^ 664, ^iii^ 689, ^iv^ 580, ^v^ 640

**Figures 1:**
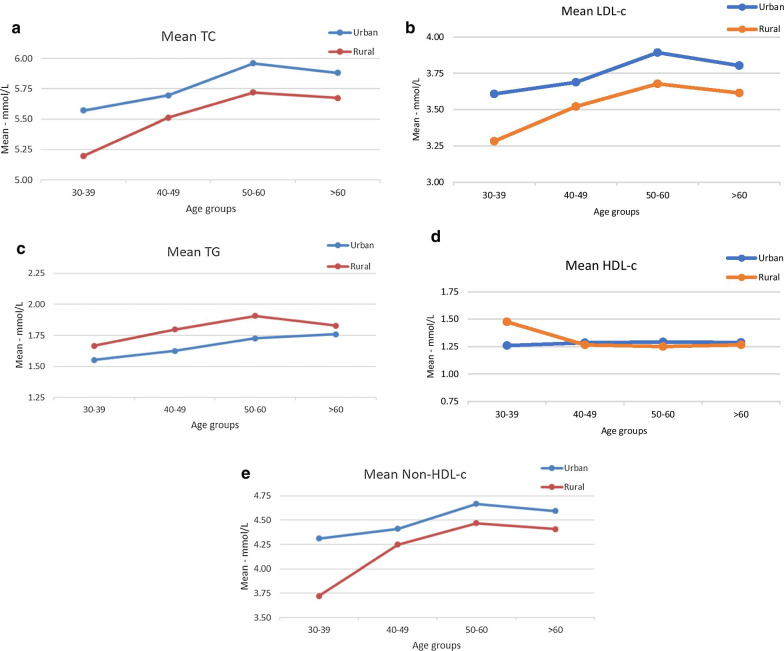
Age-specific difference of mean values of lipid profiles between urban and rural Malaysian adults: **a** TC, **b** LDL-c, **c** TG, **d** HDL-c and **e** Non-HDL-c

Figure 1a–e show the age-specific difference of mean values of lipid profiles between urban and rural Malaysian adults. The mean TC, LDL-c and non-HDL-c levels were consistently higher in the urban participants compared to rural participants across all age groups. In contrast, the mean TG levels were consistently higher in the rural participants compared to the urban ones across all age groups.

### Prevalence of dyslipidaemia subtypes

**Fig. 2:**
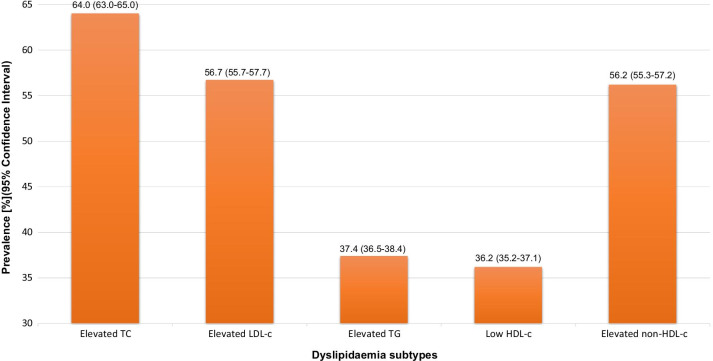
Overall prevalence of elevated TC & dyslipidaemia subtypes

The overall prevalence of elevated TC, elevated LDL-c, elevated TG, and low HDL-c were 64.0% (CI 63.0–65.0), 56.7% (CI 55.7–57.7), 37.4% (CI 36.5–38.4) and 36.2% (CI 35.2–37.1), respectively. The overall prevalence of elevated non-HDL-c was 56.2% (CI 55.3–57.2) (Fig. 2). There was no difference between the crude and age-adjusted prevalence.

Elevated TC and elevated LDL-c were more prevalent among Malays, urban participants, those with hypertension, obese, and centrally obese participants (Tables 2 and 3). Elevated TG was more prevalent among males (46.2%), rural participants (40.4%), those with diabetes (55.8%), hypertension (44.6%), obese (74.7%), and centrally obese (49.0%) (Table 4).

**Table 2:** Mean TC and prevalence of elevated TC according to personal and clinical attributes

	Mean (± SD)		Elevated TC		
		*p* value	No n (%)	Yes n (%)	*p* value
*Age groups (years)* 30–39 40–49 50–59 ≥ 60	5.39 (± 1.15) 5.61 (± 1.14) 5.85 (± 1.28) 5.76 (± 1.29)	< 0.001**	516 (45.5) 1177 (38.5) 979 (31.0) 948 (35.1)	619 (54.5) 1880 (61.5) 2177 (69.0) 1751 (64.9)	< 0.001**
**Gender** Male Female	5.68 (± 1.24) 5.72 (± 1.23)	0.129	1564 (36.2) 2056 (35.9)	2753 (63.8) 3674 (64.1)	0.719
*Ethnicity* Malay Chinese Indian Indigenous	5.89 (± 1.21) 5.54 (± 1.10) 5.51 (± 1.12) 4.90 (± 1.15)	< 0.001**	2178 (29.8) 406 (40.1) 110 (40.4) 926 (64.0)	5136 (70.2) 607 (59.9) 162 (59.6) 522 (36.1)	< 0.001**
*Education attainment* No formal education Primary Secondary Tertiary	5.51 (± 1.34) 5.77 (± 1.26) 5.75 (± 1.19) 5.72 (± 1.11)	< 0.001**	640 (44.0) 860 (34.6) 1181 (33.9) 581 (34.1)	814 (56.0) 1627 (65.4) 2302 (66.1) 1125 (65.9)	< 0.001**
*Location* Urban Rural	5.81 (± 1.16) 5.59 (± 1.30)	< 0.001**	1597 (31.7) 2023 (40.4)	3437 (68.3) 2990 (59.6)	< 0.001**
*Smoking status* Non-smoker Previous smoker Current smoker	5.71 (± 1.21) 5.69 (± 1.27) 5.71 (± 1.27)	0.830	2577 (35.9) 366 (35.0) 441 (36.0)	4611 (64.2) 681 (65.0) 784 (64.0)	0.839
*Diabetes mellitus* No Yes	5.67 (± 1.22) 5.86 (± 1.31)	< 0.001**	3127 (36.7) 493 (32.1)	5386 (63.3) 1041 (67.9)	0.001*
*Hypertension* No Yes	5.60 (± 1.20) 5.82 (± 1.27)	< 0.001**	2101 (39.0) 1519 (32.6)	3290 (61.0) 3137 (67.4)	< 0.001**
*Body mass index* Underweight Normal Overweight Obese	5.20 (± 1.12) 5.47 (± 1.21) 5.79 (± 1.23) 5.87 (± 1.20)	< 0.001**	202 (54.5) 1005 (43.9) 1221 (33.3) 962 (30.0)	169 (45.6) 1283 (56.1) 2451 (66.8) 2250 (70.1)	< 0.001**
*Waist-hip ratio* Normal Abdominal obesity	5.60 (± 1.16) 5.83 (± 1.27)	< 0.001**	1818 (39.0) 1660 (32.9)	2847 (61.0) 3379 (67.1)	< 0.001**

^*^significant at *p* < 0.05; **significant at *p* < 0.001

**Table 3:** Mean LDL-c and prevalence of elevated LDL-c according to personal and clinical attributes

	Mean (± SD)		Elevated LDL-c		
		*p* value	No n (%)	Yes n (%)	*p* value
*Age groups (years)* 30–39 40–49 50–59 ≥ 60	3.45 (± 1.01) 3.61 (± 1.03) 3.79 (± 1.14) 3.69 (± 1.12)	< 0.001**	570 (50.3) 1368 (44.8) 1242 (39.4) 1169 (43.3)	564 (49.7) 1689 (55.3) 1910 (60.6) 1529 (56.7)	< 0.001**
*Gender* Male Female	3.69 (± 1.10) 3.66 (± 1.09)	0.245	1817 (42.1) 2532 (44.2)	2497 (57.9) 3195 (55.8)	0.036*
*Ethnicity* Malay Chinese Indian Indigenous	3.84 (± 1.07) 3.42 (± 0.98) 3.55 (± 1.00) 3.02 (± 1.01)	< 0.001**	2696 (36.9) 521 (51.6) 125 (46.0) 1007 (69.5)	4616 (63.1) 488 (48.4) 147 (54.0) 441 (30.5)	< 0.001**
*Education attainment* No formal education Primary Secondary Tertiary	3.49 (± 1.15) 3.72 (± 1.12) 3.72 (± 1.07) 3.70 (± 1.00)	< 0.001**	753 (51.8) 1053 (42.3) 1428 (41.1) 696 (40.8)	700 (48.2) 1434 (57.7) 2050 (58.9) 1010 (59.2)	< 0.001**
*Location* Urban Rural	3.77 (± 1.04) 3.57 (± 1.13)	< 0.001**	1954 (38.9) 2395 (47.8)	3076 (61.2) 2616 (52.2)	< 0.001**
*Smoking status* Non-smoker Previous smoker Current smoker	3.67 (± 1.08) 3.71 (± 1.13) 3.70 (± 1.09)	0.456	3131 (43.6) 448 (42.8) 502 (41.0)	4052 (56.4) 599 (57.2) 722 (59.0)	0.235
*Diabetes mellitus* No Yes	3.67 (± 1.09) 3.69 (± 1.13)	0.479	3704 (43.5) 645 (42.1)	4804 (56.5) 888 (57.9)	0.288
*Hypertension* No Yes	3.62 (± 1.05) 3.74 (± 1.14)	< 0.001**	2455 (45.6) 1894 (40.7)	2934 (54.4) 2758 (59.3)	< 0.001**
*Body mass index* Underweight Normal Overweight Obese	3.20 (± 0.96) 3.45 (± 1.06) 3.76 (± 1.11) 3.80 (± 1.06)	< 0.001**	244 (65.8) 1198 (52.4) 1468 (40.0) 1201 (37.4)	127 (34.2) 1089 (47.6) 2202 (60.0) 2008 (62.6)	< 0.001**
*Waist-hip ratio* Normal Abdominal obesity	3.59 (± 1.04) 3.76 (± 1.13)	< 0.001**	2061 (46.6) 1814 (39.9)	2364 (53.4) 2737 (60.1)	< 0.001**

^*^significant at *p* < 0.05; **significant at *p* < 0.001

**Table 4:** Mean TG and prevalence of elevated TG according to personal and clinical attributes

	Mean (± SD)		Elevated TG		
		*p* value	No n (%)	Yes n (%)	*p* value
*Age groups (years)* 30–39 40–49 50–59 ≥ 60	1.61 (± 1.05) 1.71 (± 1.07) 1.81 (± 1.07) 1.80 (± 0.96)	< 0.001**	792 (70.2) 1995 (65.4) 1874 (59.4) 1617 (59.9)	336 (29.8) 1056 (34.6) 1279 (40.6) 1082 (40.1)	< 0.001**
*Gender* Male Female	1.95 (± 1.14) 1.60 (± 0.93)	< 0.001**	2319 (53.8) 3959 (69.2)	1992 (46.2) 1761 (30.8)	< 0.001**
*Ethnicity* Malay Chinese Indian Indigenous	1.79 (± 1.05) 1.52 (± 1.01) 1.77 (± 1.12) 1.70 (± 0.98)	< 0.001**	4427 (60.7) 730 (72.1) 173 (63.6) 948 (65.5)	2871 (39.3) 283 (27.9) 99 (36.4) 500 (34.5)	< 0.001**
*Education attainment* No formal education Primary Secondary Tertiary	1.80 (± 0.99) 1.82 (± 1.02) 1.72 (± 1.01) 1.65 (± 1.12)	< 0.001**	873 (60.0) 1487 (59.8) 2227 (64.0) 1147 (67.3)	581 (40.0) 1000 (40.2) 1255 (36.0) 558 (32.7)	< 0.001**
*Location* Urban Rural	1.68 (± 1.04) 1.82 (± 1.04)	< 0.001**	3298 (65.6) 2980 (59.6)	1733 (34.5) 2020 (40.4)	< 0.001**
*Smoking status* Non-smoker Previous smoker Current smoker	1.68 (± 0.98) 1.84 (± 1.05) 2.08 (± 1.22)	< 0.001**	4727 (65.8) 597 (57.0) 603 (49.3)	2459 (34.2) 450 (43.0) 621 (50.7)	< 0.001**
*Diabetes mellitus* No Yes	1.67 (± 0.95) 2.23 (± 1.34)	< 0.001**	5601 (65.9) 677 (44.2)	2898 (34.1) 855 (55.8)	< 0.001**
*Hypertension* No Yes	1.63 (± 1.00) 1.89 (± 1.07)	< 0.001**	3700 (68.8) 2578 (55.4)	1675 (31.2) 2078 (44.6)	< 0.001**
*Body mass index* Underweight Normal Overweight Obese	1.33 (± 0.70) 1.49 (± 0.87) 1.76 (± 1.02) 1.97 (± 1.13)	< 0.001**	307 (82.8) 1717 (75.1) 2271 (61.9) 1681 (52.3)	64 (17.3) 570 (24.9) 1399 (38.1) 1531 (47.7)	< 0.001**
*Waist-hip ratio* Normal Abdominal obesity	1.50 (± 0.82) 2.00 (± 1.16)	< 0.001**	3302 (74.6) 2321 (51.0)	1123 (25.4) 2232 (49.0)	< 0.001**

^*^significant at *p* < 0.05; **significant at *p* < 0.001

For low HDL-c, Indigenous participants (52%), current smokers (45.7%), participants with diabetes (44.1%), hypertension (37.4%), obesity (45.6%), and central obesity (43.8%) had higher prevalence compared to the others (Table 5). Elevated non-HDL-c was more prevalent in males (60.8%), Malays (62.5%), urban participants (58.5%), those with diabetes (64.5%), hypertension (61.3%), obese (65.6%), and centrally obese (63.6%) (Table 6).

**Table 5:** Mean HDL-c and prevalence of low HDL-c according to personal and clinical attributes

	Mean (± SD)		Low HDL-c		
		*p* value	No n (%)	Yes n (%)	*p* value
*Age groups (years)* 30–39 40–49 50–59 ≥ 60	1.36 (± 0.76) 1.28 (± 0.50) 1.27 (± 0.51) 1.28 (± 0.47)	< 0.001**	714 (62.9) 1920 (62.8) 1997 (63.3) 1784 (66.1)	421 (37.1) 1137 (37.2) 1159 (36.7) 915 (33.9)	0.041*
*Gender* Male Female	1.15 (± 0.46) 1.39 (± 0.56)	< 0.001**	2731 (63.3) 3684 (64.3)	1586 (36.7) 2046 (35.7)	0.287
*Ethnicity* Malay Chinese Indian Indigenous	1.26 (± 0.42) 1.50 (± 0.65) 1.17 (± 0.35) 1.26 (± 0.86)	< 0.001**	4743 (64.9) 820 (81.0) 157 (57.7) 695 (48.0)	2571 (35.2) 193 (19.1) 115 (42.3) 753 (52.0)	< 0.001**
*Education attainment* No formal education Primary Secondary Tertiary	1.25 (± 0.60) 1.26 (± 0.50) 1.29 (± 0.53) 1.31 (± 0.47)	0.004*	806 (55.4) 1558 (62.7) 2234 (64.1) 1200 (70.3)	648 (44.6) 929 (37.4) 1249 (35.9) 506 (29.7)	< 0.001**
*Location* Urban Rural	1.29 (± 0.38) 1.28 (± 0.65)	0.964	3407 (67.7) 3008 (60.0)	1627 (32.3) 2005 (40.0)	< 0.001**
*Smoking status* Non-smoker Previous smoker Current smoker	1.32 (± 0.52) 1.19 (± 0.47) 1.08 (± 0.37)	< 0.001**	4680 (65.1) 667 (63.7) 665 (54.3)	2508 (34.9) 380 (36.3) 560 (45.7)	< 0.001**
*Diabetes mellitus* No Yes	1.30 (± 0.54) 1.19 (± 0.45)	< 0.001**	5557 (65.3) 858 (55.9)	2956 (34.7) 676 (44.1)	< 0.001**
*Hypertension* No Yes	1.30 (± 0.52) 1.27 (± 0.54)	< 0.001**	3501 (64.9) 2914 (62.6)	1890 (35.1) 1742 (37.4)	0.014*
*Body mass index* Underweight Normal Overweight Obese	1.46 (± 0.63) 1.40 (± 0.64) 1.27 (± 0.50) 1.19 (± 0.44)	< 0.001**	269 (72.5) 1700 (74.3) 2375 (64.7) 1749 (54.5)	102 (27.5) 588 (25.7) 1297 (35.3) 1463 (45.6)	< 0.001**
*Waist-hip ratio* Normal Abdominal obesity	1.39 (± 0.57) 1.19 (± 0.47)	< 0.001**	3177 (71.8) 2559 (56.2)	1250 (28.2) 1995 (43.8)	< 0.001**

^*^significant at *p* < 0.05; **significant at *p* < 0.001

**Table 6:** Mean non-HDL-c and prevalence of elevated non-HDL-c according to personal and clinical attributes

	Mean (± SD)		Elevated non-HDL-c		
		*p* value	No n (%)	Yes n (%)	*p* value
*Age groups (years)* 30–39 40–49 50–59 ≥ 60	4.03 (± 1.52) 4.33 (± 1.28) 4.58 (± 1.38) 4.48 (± 1.33)	< 0.001**	607 (53.5) 1410 (46.1) 1231 (39.0) 1153 (42.7)	528 (46.5) 1647 (53.9) 1925 (61.0) 1546 (57.3)	0.000*
*Gender* Male Female	4.53 (± 1.33) 4.33 (± 1.38)	< 0.001**	1693 (39.2) 2708 (47.3)	2624 (60.8) 3022 (52.7)	< 0.001**
*Ethnicity* Malay Chinese Indian Indigenous	4.62 (± 1.27) 4.04 (± 1.31) 4.34 (± 1.15) 3.65 (± 1.52)	< 0.001**	2740 (37.5) 566 (55.9) 128 (47.1) 967 (66.8)	4574 (62.5) 447 (44.1) 144 (52.9) 481 (33.2)	< 0.001**
*Education attainment* No formal education Primary Secondary Tertiary	4.26 (± 1.44) 4.51 (± 1.35) 4.46 (± 1.33) 4.41 (± 1.22)	< 0.001**	724 (49.8) 1006 (40.5) 1492 (42.8) 746 (43.7)	730 (50.2) 1481 (59.6) 1991 (57.2) 960 (56.3)	< 0.001**
*Location* Urban Rural	4.53 (± 1.19) 4.30 (± 1.51)	< 0.001**	2088 (41.5) 2313 (46.1)	2946 (58.5) 2700 (53.9)	0.002*
*Smoking status* Non-smoker Previous smoker Current smoker	4.39 (± 1.32) 4.51 (± 1.36) 4.63 (± 1.30)	< 0.001**	3246 (45.2) 418 (39.9) 457 (37.3)	3942 (54.8) 629 (60.1) 768 (62.7)	< 0.001**
*Diabetes mellitus* No Yes	4.37 (± 1.35) 4.67 (± 1.37)	< 0.001**	3857 (45.3) 544 (35.5)	4656 (54.7) 990 (64.5)	< 0.001**
*Hypertension* No Yes	4.30 (± 1.33) 4.55 (± 1.38)	< 0.001**	2598 (48.2) 1803 (38.7)	2793 (51.8) 2853 (61.3)	< 0.001**
*Body mass index* Underweight Normal Overweight Obese	3.73 (± 1.21) 4.06 (± 1.37) 4.52 (± 1.33) 4.67 (± 1.27)	< 0.001**	260 (70.1) 1301 (56.9) 1482 (40.4) 1105 (34.4)	111 (29.9) 987 (43.1) 2190 (59.6) 2107 (65.6)	< 0.001**
*Waist-hip ratio* Normal Abdominal obesity	4.21 (± 1.31) 4.64 (± 1.34)	< 0.001**	2261 (51.1) 1659 936.4)	2166 (48.9) 2895 (63.6)	< 0.001**

^*^significant at *p* < 0.05; **significant at *p* < 0.001

### Personal and clinical attributes associated with dyslipidaemia subtypes

Table 7 displays a multiple logistic regression model used to ascertain the personal and clinical attributes associated with the subtypes of dyslipidaemia. Compared to the 30 to 39 age group, those in the 50 to 59 age group were associated with higher adjusted odds ratio for all subtypes of dyslipidaemia except for elevated TG, and lower adjusted odds ratio for low HDL-c. Females had increased likelihood of low HDL-c [aOR 1.14 (95% CI 1.02–1.29)], but reduced likelihood of high TG [aOR 0.62 (95% CI 0.55–0.70)] and high non-HDL-c [aOR 0.79 (95% CI 0.71–0.88)], compared to males.

**Table 7:** Personal and clinical attributes associated with subtypes of dyslipidaemia

	High TC	High LDL-c	High TG	Low HDL-c	High Non-HDL-c
	aOR (95% CI)	aOR (95% CI)	aOR (95% CI)	aOR (95% CI)	aOR (95% CI)
*Age (years)* 30–39 40–49 50–59 ≥ 60	1.00 **1.28 (1.09–1.50)** **1.78 (1.50–2.10)** **1.58 (1.31–1.90)**	1.00 **1.25 (1.07–1.46)** **1.54 (1.31–1.82)** **1.42 (1.18–1.71)**	1.00 1.05 (0.88–1.25) 1.15 (0.96–1.38) 1.00 (0.82–1.21)	1.00 0.93 (0.78–1.09) **0.81 (0.68–0.96**) **0.67 (0.55–0.81)**	1.00 **1.21 (1.03–1.42)** **1.50 (1.27–1.77)** **1.30 (1.08–1.57)**
**Gender** Male Female	1.00 1.12 (1.00–1.26)	1.00 0.98 (0.87–1.09)	1.00 **0.62 (0.55–0.70)**	1.00 **1.14 (1.02–1.29)**	1.00 **0.79 (0.71–0.88)**
**Ethnicity** Malay Chinese Indian Indigenous	1.00 **0.70 (0.60–0.83)** **0.60 (0.45–0.80)** **0.27 (0.23–0.32)**	1.00 **0.58 (0.49–0.68)** **0.61 (0.46–0.81)** **0.29 (0.25–0.34)**	1.00 0.92 (0.77–1.11) 0.90 (0.66–1.23) 0.88 (0.75–1.03)	1.00 **0.55 (0.45–0.67)** 1.28 (0.95–1.72) **2.42 (2.08–2.81)**	1.00 **0.60 (0.51–0.71)** **0.63 (0.47–0.84)** **0.33 (0.28–0.39)**
**Educational attainment** No formal Primary Secondary Tertiary	1.00 1.06 (0.91–1.24) 1.07 (0.91–1.26) 1.04 (0.85–1.27)	1.00 1.04 (0.90–1.21) 1.02 (0.87–1.20) 0.99 (0.81–1.20)	1.00 **0.78 (0.67–0.91)** **0.76 (0.65–0.90)** **0.75 (0.62–0.92)**	1.00 **0.76 (0.65–0.88)** **0.71 (0.61–0.84)** **0.60 (0.49–0.73)**	1.00 0.98 (0.84–1.14) 0.88 (0.75–1.04) 0.86 (0.71–1.05)
**Location** Urban Rural	1.00 0.95 (0.85–1.07)	1.00 **0.84 (0.75–0.95)**	1.00 **1.39 (1.24–1.57)**	1.00 0.99 (0.88–1.11)	1.00 1.01 (0.90–1.13)
**Smoking** Non-smoker Previous smoker Current smoker	1.00 1.03 (0.87–1.22) 1.06 (0.91–1.25)	1.00 1.00 (0.85–1.17) 1.14 (0.98–1.33)	1.00 1.04 (0.88–1.22) **1.49 (1.27–1.74)**	1.00 1.11 (0.95–1.31) **1.76 (1.50–2.06)**	1.00 1.01 (0.86–1.19) **1.25 (1.06–1.46)**
**Body mass index** Normal Underweight Overweight Obese	1.00 **0.61 (0.48–0.78)** **1.36 (1.20–1.54)** **1.36 (1.19–1.56)**	1.00 **0.55 (0.43–0.71)** **1.45 (1.29–1.64)** **1.43 (1.26–1.63)**	1.00 **0.72 (0.53–0.98)** **1.67 (1.46–1.91)** **2.34 (2.03–2.70)**	1.00 1.04 (0.79–1.37) **1.65 (1.44–1.89)** **2.51 (2.17–2.89)**	1.00 **0.53 (0.41–0.69)** **1.66 (1.47–1.87)** **1.91 (1.67–2.18)**
**Waist-hip ratio** Normal Abdominal obesity	1.00 **1.17 (1.05–1.30)**	1.00 **1.12 (1.01–1.23)**	1.00 **1.86 (1.67–2.06)**	1.00 **1.62 (1.46–1.80)**	1.00 **1.33 (1.21–1.48)**
**Diabetes mellitus** No Yes	1.00 0.89 (0.78–1.02)	1.00 **0.78 (0.69–0.89)**	1.00 **1.90 (1.67–2.16)**	1.00 **1.34 (1.18–1.53)**	1.00 1.06 (0.93–1.21)
**Hypertension** No Yes	1.00 **1.16 (1.04–1.28)**	1.00 1.10 (1.00–1.21)	1.00 **1.36 (1.23–1.51)**	1.00 0.95 (0.85–1.05)	1.00 **1.20 (1.09–1.33)**

Multivariable regression model, controlled for age, gender, ethnicity, educational attainment, locality, smoking status, BMI, waist-hip ratio, diabetes mellitus status, hypertension status. No significant interactions

The Indigenous population had lower odds for elevated TC [aOR 0.27 (95% CI 0.23–0.32)], elevated LDL-c [aOR 0.29 (95% CI 0.25–0.34)], and elevated non-HDL-c [aOR 0.33 (95% CI 0.28–0.39)], compared to Malays. However, they had almost 2.5 times [aOR 2.42 (95% CI 2.08–2.81)] increased odds of having low HDL-c. Compared to urban participants, rural participants had increased likelihood of having high TG [aOR 1.39 (95% CI 1.24–1.57)], but they were less likely to have high LDL-c [aOR 0.84 (95% CI 0.75–0.95)].

Overweight, obesity and central obesity were significantly associated with elevated TC and all subtypes of dyslipidaemia compared to normal BMI and normal waist-hip ratios. In contrast, participants who were underweight were associated with reduced odds for elevated TC and all dyslipidaemia subtypes except for low HDL-c.

Participants with hypertension had increased adjusted OR for elevated TC, elevated TG, and elevated non-HDL-c. Participants with diabetes had increased likelihood of elevated TG [aOR 1.90 (95% CI 1.67–2.16)] and reduced HDL-c [aOR 1.34 (95% CI 1.18–1.53)].

## Discussion

The REDISCOVER Study found that almost two-thirds of participants have elevated TC. In comparison with the National Health and Morbidity Survey 2011 (NHMS 2011) which included participants above 18 years old, the prevalence of hypercholesterolaemia in our study was nearly twice as high (64.0% vs. 35.1%) [10]. The national prevalence of hypercholesterolaemia has shown an increasing trend by the year 2015 (47.7%) [11]. In a recent review article, Lin et al*.* reported the wide range of hypercholesterolaemia prevalence in the Asia Pacific region [8]. Comparing with other South East Asian countries, the prevalence of hypercholesterolaemia were lower at 46.9% in the Philippines and 35.8% in Indonesia [7, 17]. The prevalence in Singapore, based on its National Health Survey 2010 which included adults 18 years and above, was comparatively lower at 17.4% [18]. However, this was based on a cut-off of ≥ 6.2 mmol/L. When the cut-off was taken at a similar level to this study (≥ 5.2 mmol/L), the prevalence was 51.5%.

In our study, the prevalence of elevated LDL-c was found to be high at 56.7%. Nawawi et al*.* studied 609 rural Malays and found a comparable prevalence of 57.2% [12]. Another smaller study involving 148 factory workers in Kelantan reported a prevalence of 38.2% [13]. This lower prevalence may be explained by the higher cut-off used (≥ 4.1 mmol/L). The prevalence of elevated LDL-c in the Philippines was 47.2% [17] and 15.2% in Singapore; both reports included adults 18 years and above, and Singapore used a higher cut-off of ≥ 4.1 mmol/L [18].

This study found the prevalence of elevated TG to be 37.4%, which is slightly lower to that reported by Nawawi et al*.* (46.1%) [12] and Nazri et al*.* (42.1%) [13]. Prevalence of this dyslipidaemia subtype was found to be similar in the Philippines and Thailand, both at 38.6% [17, 19]. For low HDL-c, the prevalence was 36.2% in this study, compared to Nawawi et al*.* (13.1%) [12] and Nazri et al*.* (9.2%) [13]. These differences could be contributed by the lower cut off (< 0.9 mmol/L for both males and females) used by Nawawi et al*.*, and the influence of dietary intake and physical activity levels [12, 13]. Thailand had a prevalence of 47.1% [19], but the Philippines had a very high of prevalence of 71.3% [17]. The reasons for this great difference is not apparent but a combination of genetic, nutritional and environmental factors had been proposed [20].

Recent evidence suggests that non-HDL-c is a more powerful CV risk predictor compared to LDL-c [2–4]. The prevalence of this dyslipidaemia subtype was 56.2% in our study. There are no previous published local studies on this subtype of dyslipidaemia. A study in Colombia reported a very high prevalence of 75.3%, making it their most prevalent dyslipidaemia subtype [21]. The authors proposed that this is due to the high refined carbohydrate intake in their population. The latest Malaysian dyslipidaemia clinical practice guidelines recommended that non-HDL-c should only be a secondary target, after the LDL-c target has been achieved for patients with combined hyperlipidaemias, diabetes, cardiometabolic risk, or chronic kidney disease [1]. Given the high prevalence rate of this highly atherogenic dyslipidaemia subtype, this recommendation may need to be revised.

Malays, who make up 51% of the population in this country [22] had the highest prevalence for elevated TC (70.2%), elevated LDL-c (63.1%), and elevated non-HDL-c (62.5%). The multiple logistic regression model showed that the other ethnicities had lower adjusted odds ratio for these three dyslipidaemia subtypes. Nawawi et al*.* found the prevalence of these dyslipidaemia subtypes in rural Malays were 67.3% (high TC) and 57.2% (high LDL-c) [12]. The 2011 National Health and Morbidity Survey also reported that Malays had the highest prevalence of high TC (38.4%) [10]. The prevalence rate might be lower because it included adults 18 years old and older. Underlying genetic predisposition, dietary habits and physical activity levels have been proposed as possible causes for the high prevalence of these dyslipidaemia subtypes in Malays [23].

Interestingly, the Indigenous population was associated with almost 2.5 times increased odds ratio to have low HDL-c [aOR 2.42 (95% CI 2.08–2.81)], compared to Malays. This is a heterogenous group composed of multiple ethnic groups, with the majority from the Kadazan-Dusun ethnic from East Malaysia. There is no previous published data for comparison. Further studies in this population are needed to confirm this observation and determine its significance especially on the occurrence of atherosclerotic cardiovascular disease.

Two measures to characterize body composition were included in this study, which were BMI and WHR. Elevated levels in each method were associated with higher odds ratio of elevated TC and all dyslipidaemia subtypes. This finding is consistent with previous studies [21]. Although both measures had significant association with increased likelihood of elevated TC and all dyslipidaemia subtypes, the adjusted OR for BMI were higher compared to WHR. This suggest that BMI has a stronger association with dyslipidaemia in our population.

### Strengths and limitations of the study

The strengths of this study include the large sample size and high response rate, strengthening the external validity of the findings. However, as a community activity where participation was on a voluntary basis, all eligible participants who attended were recruited, resulting in the total number of participants recruited exceeding the calculated sample size. Given the voluntary nature of participating in this study, there is a possibility that the participants were more health-conscious than the general population. Malays were over-represented in this study while Chinese and Indians were under-represented. Our study used the Malaysian guidelines as the cut-off points for dyslipidaemia. Therefore, this might affect direct comparisons with studies utilizing other guidelines. Finally, this study looked at the cross-sectional baseline data hence the findings can only show association but not causality. Therefore, interpretation and generalization of the results should be done with care.

### Implications for clinical practice and future research

REDISCOVER provided important insights on the current magnitude and associated factors of dyslipidaemia among Malaysians. Knowing the high prevalence of these dyslipidaemia subtypes may raise the clinicians’ awareness of this issue and ensure a holistic approach to managing dyslipidaemia. Laboratory reporting of non-HDL-c as part of the lipid profile should also be introduced to raise awareness of its importance among clinicians. Given the high prevalence of dyslipidaemia, immediate actions are needed now. These should include opportunistic screening for all 30 years old and older, Malays, those who are overweight or centrally obese, and those with hypertension or diabetes. As obesity has been consistently shown to be associated with CVD and its risk factors, more effective public health measures need to be implemented to educate all Malaysians regarding the importance of healthy and balanced dietary intake, and adequate physical activity levels to address this epidemic. Further studies involving dietary intake and physical activity among Malaysians, as well as looking into the association between the Indigenous population with low HDL-c levels may provide valuable information on dyslipidaemia in this country. More research to establish the relationship between elevated non-HDL-c and atherosclerotic CVD in Malaysians is also needed. All these will form the evidence-base for future local dyslipidaemia management guidelines and practice.

## Conclusions

The REDISCOVER Study found that the prevalence of elevated TC and all dyslipidaemia subtypes is worryingly high in Malaysian adults, where increased body mass seems to be the main driver. Differences of the dyslipidaemia subtype prevalence between personal and clinical attributes of individuals observed may have specific use in the realm of precision medicine such that a more targeted approach may be employed in the prevention and treatment of dyslipidaemia.

## Data Availability

Data are kept at the Centre for Translational Research and Epidemiology (CenTRE), Faculty of Medicine, Universiti Teknologi MARA. Data will be shared upon request and it is subjected to the data protection regulation.

## Abbreviations

aOR: Adjusted odds ratio
BMI: Body mass index
BP: Blood pressure
CI: Confidence interval
CVD: Cardiovascular diseases
HDL-c: High-density lipoprotein cholesterol
LDL-c: Low-density lipoprotein cholesterol
NHMS: National Health and Morbidity Survey
Non-HDL-c: Non-high-density lipoprotein cholesterol
OR: Odds ratio
SD: Standard deviation
TC: Total cholesterol
TG: Triglycerides
VLDL: Very low-density lipoprotein
WHR: Waist-hip ratio
